# Familial segregation of a *VSX1* mutation adds a new dimension to its role in the causation of keratoconus

**Published:** 2011-02-15

**Authors:** Preeti Paliwal, Radhika Tandon, Divya Dube, Punit Kaur, Arundhati Sharma

**Affiliations:** 1 Laboratory of Cyto-Molecular Genetics, Department of Anatomy, All India Institute of Medical Sciences, New Delhi, India; 2 Dr. Rajendra Prasad Centre for Ophthalmic Sciences, All India Institute of Medical Sciences, New Delhi, India; 3 Department of Biophysics, All India Institute of Medical Sciences, New Delhi, India

## Abstract

**Purpose:**

To look for segregation of Visual System Homeobox 1 (*VSX1*) mutations in family members of a patient with keratoconus.

**Methods:**

Our initial molecular genetic studies conducted to identify the role of *VSX1* in the causation of keratoconus had identified a novel mutation in one patient. He later presented to the clinic affected with vernal kerato conjunctivitis (VKC) accompanied by his brother, also similarly affected. All the family members were called and detailed clinical evaluations were undertaken. DNA from the blood samples of all family members was amplified using primers specific for *VSX1* and analyzed by direct sequencing to look for segregation of the mutation in the family members. Protein modeling studies were done to assess the effect of the mutation on protein structure and function.

**Results:**

Clinical examination of the family revealed bilateral keratoconus and VKC in the proband and his brother. One of his sisters had VKC without keratoconus and his parents and another sister were normal. Molecular analysis identified the VSX1 mutation Q175H in the affected brother and in the mother who had neither VKC nor keratoconus but only the VSX1 Q175H sequence change.

**Conclusions:**

The VSX1 Q175H mutation may be a pathogenic variant with incomplete penetrance. Protein modeling studies show that the mutation affects the DNA binding properties of the protein. This *VSX1* variant exhibiting low penetrance may require the presence of some modifier genes or environmental factors for disease presentation. *VSX1* may have an important role in the pathogenesis of keratoconus which needs further investigation.

## Introduction

Keratoconus (KC; OMIM 148300) is a bilateral, progressive corneal ectasia characterized by protrusion of the apical cornea due to thinning, leading to progressive myopia and irregular astigmatism [1]. Severely affected patients often require corneal transplantation. The pathophysiology of keratoconus is not completely understood, though various studies have suggested keratoconus to be associated with eye rubbing [2,3] and use of contact lenses [4]. The other commonly associated ocular disorders include vernal keratoconjunctivitis [5], retinitis pigmentosa [6], and Leber congenital amaurosis (LCA) [7]. Various connective tissue disorders like Ehlers-Danlos and Marfan syndromes are also known to be associated with keratoconus [8].

Vernal keratoconjunctivitis (VKC) is the inflammation of cornea and conjunctiva, usually occurring during spring season. It is considered to be caused by allergens but the role of several genes is also implicated [9]. A higher incidence of keratoconus is seen in patients affected with VKC where eye rubbing is suggested as a possible cause of keratoconus [10].

Several chromosomal loci and genes are reported to be associated with the occurrence of keratoconus [11]. One of the widely studied genes is the Visual System Homeobox 1 (*VSX1*; OMIM 605020) localized to chromosome 20p11-q11. The true association of this gene with KC is not yet established due to the conflicting reports of the various studies which have been conducted world over [11-13].

We reported a mutation Q175H in *VSX1* in an individual diagnosed with keratoconus. This change was not seen in 100 control individuals who were also analyzed for the underlying changes in *VSX1* [14]. In silico analysis of the change using SIFT performed previously had given a score of <0.05 which showed that the change was not tolerated and the glutamine at position 175 is conserved across species [14]. We subsequently contacted the patient for further detailed study of the family. We now report the segregation of this change (Q175H) in the family members of the proband.

## Methods

The study adhered to the tenets of Declaration of Helsinki and was approved by the Institutional Ethics Committee of All India Institute of Medical Sciences (AIIMS). The proband initially presented in the Cornea and Refractive Surgeries Services and Contact Lens Clinic at Dr. Rajendra Prasad Centre for Ophthalmic Sciences, All India Institute of Medical Sciences two years back at 19 years of age with diminution of vision for past 8 years and recurrent redness and watering. He was diagnosed to have KC and molecular analysis revealed a novel *VSX1* mutation in him. He presented to the clinic again after 2 years with his brother aged 18 years, also affected with diminution of vision, recurrent redness and watering. Detailed family history up to three generations was taken and other family members (parents and two sisters) were called to the clinic. Informed consent was taken from all the participants after explaining the nature and possible consequences of study participation. Detailed clinical examination was performed for identifying and characterizing keratoconus in the family members that included corneal stromal thinning, Vogt’s striae, Fleischer’s ring, Munson’s sign, and corneal topography, i.e., corneal power (K), inferior–superior dioptric asymmetry (I-S), astigmatism (Ast), and skewed radial axis (SRAX) using Orbscan II operating with software version 3.12 (Bausch & Lomb Inc., Rochester, NY) that were used to calculate KISA% (a single index that quantifies the irregular corneal shape and astigmatism typical of keratoconus with good clinical correlation) according to Massachusetts Ear and Eye Infirmary KC Classification.

### DNA extraction and PCR amplification

A total of 5 ml peripheral blood samples were drawn from the affected and unaffected family members and genomic DNA was extracted from peripheral blood leukocytes using standard protocols. All the five exons and intron/exon boundaries of *VSX1* were amplified using custom-synthesized oligonucleotide primers as described previously [14] and presented in Table 1. Each reaction was performed in a 25 μl mixture containing 2.5 μl 10× PCR buffer with 3.5 mM MgCl_2_, 2.5 mM dNTPs, 10 pM of each primer, 0.7 U Taq DNA polymerase (Roche, Applied Biosystems, Foster City CA) and 100 ng genomic DNA. Thermal cycling was performed in a thermal cycler (Applied Biosystem 9700; Roche, Applied Biosystems) as described herein: initial denaturation for 12 min at 95 °C; 35 cycles of 94 °C for 30 s, 62 °C (exons 1–4) or 60 °C (exon 5) for 30 s, 72 °C for 30 s, and a final extension for 10 min at 72 °C.

**Table 1:** The *VSX1* primer sequences.

**Exon**	**Forward Primer**	**Reverse Primer **	**Amplicon size (bp)**	**Melting temperature**
1	5’-CAGCTGATTGGAGCCCTTC-3’	5’-CTCAGAGCCTAGGGGACAGG-3’	599	58 °C
2	5’-GCCCACTAAAAATGCAGAA-3’	5’-GCCTCCTAGGAACTGCAGAA-3’	393	59 °C
3	5’-CATTCAGAGGTGGGGTGTT-3’	5’-TCTTGTGGTGCCTTCAGCTA-3’	419	62 °C
4	5’-GATCATGATCGGGAGAGAAG-3’	5’-CGTTGCTTTGCTTTGGAAAT-3’	394	59 °C
5	5’-CCCCAGAGATAGGCACTGAC-3’	5’-TGGACAATTTTTGTCTTTTGG-3’	495	59 °C

### Sequencing

All the amplified products were sequenced bidirectionally using BigDye Terminator Mix version 3.1 (Applied Biosystems [ABI], Foster City, CA) according to the manufacturer’s instructions and were analyzed on an ABI-3100 Genetic Analyzer (ABI). Nucleotide sequences were compared with the published *VSX1* cDNA sequence (GenBank NM_014588).

### Homology modeling

Protein homology modeling and Molecular Dynamics (MD) simulations was performed for the VSX1 Q175H change to assess the structural implications of this mutation on the protein conformation. VSX1 has 58% sequence identity with the homeodomain of *Drosophila*. A homology model was built using the three-dimensional X-ray crystal structure of homeodomain (Protein Data Base code: 1FJL) as a template with the model building software Modeller9v7. Mutant VSX1 (Q175H) was generated by altering corresponding residue and the resulting mutated structures were studied by applying molecular dynamics simulation using DS 2.0 (Messrs Accelrys, San Diego, CA). The structures of the native protein and mutants were validated for their accuracy and correctness using PROCHECK [15] and figures were drawn with PYMOL (Molecular Graphics System [2002]; DeLano *S*cientific, San Carlos, CA).

### Molecular dynamics simulation

To check the effect of the change on the overall conformation of VSX1, extensive MD simulations were performed on a fully hydrated model of both wild type (wVSX1) and mutant (mVSX1). The structures of both wild-type and mutant were further analyzed for differences induced in the protein conformation due to the change.

## Results

**Figure 1:**
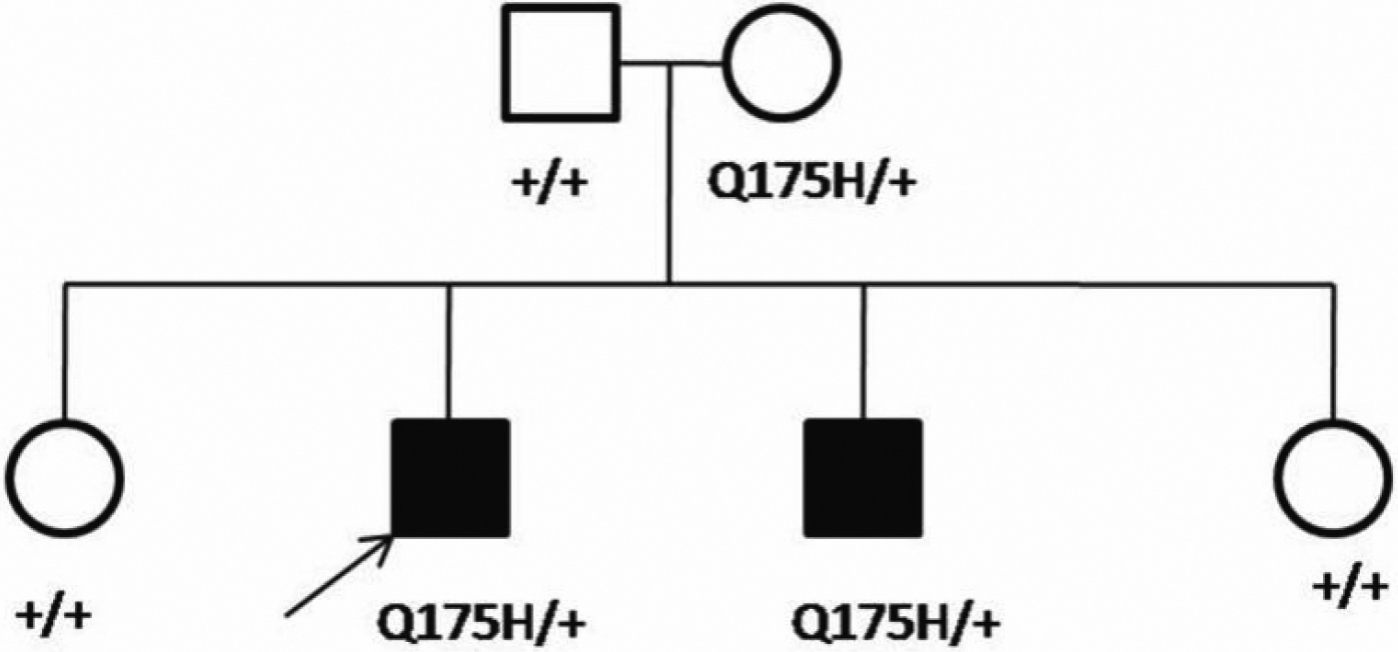
Pedigree of the family showing the heterozygous *VSX1* c.525G>C (Q175H) nucleotide change. Filled symbols represent individuals affected with keratoconus and open symbols represent unaffected individuals. The arrow indicates the proband, the sign ‘+’ represents the wild type and Q175H is the mutation detected.

Clinical examination of the proband who had presented at 19 years of age with diminution of vision for past eight years, recurrent redness, itching and watering had revealed bilateral keratoconus (corneal pachymetry- OD-432, OS-448; simulated keratometer value-OD-55.70D, OS-51.00D). Clinical evaluation on his successive visit revealed presence of VKC. His brother, aged 18 years was also found to be affected with keratoconus (corneal pachymetry- OD-452, OS-460; simulated keratometer value- OD-48.50D, OS-55.85D) and VKC. Another sister who was 15 years old had VKC but no keratoconus (corneal pachymetry- OD-544, OS-539; simulated keratometer value- OD-45.20D, OS-45.00D). The parents (father 55 years and mother 50 years) and one sister aged 23 revealed a disease free cornea (Figure 1). Mutational analysis of *VSX1* was performed in all the family members. The heterozygous change c.525G>C identified in the patient was seen in the affected brother and the clinically unaffected mother (The clinical examinations of mother revealed corneal pachymetry- OD-560, OS-562; simulated keratometer value- OD-46.50D, OS-47.00D). The father and the two sisters did not reveal this change.

### Protein modeling

**Figure 2:**
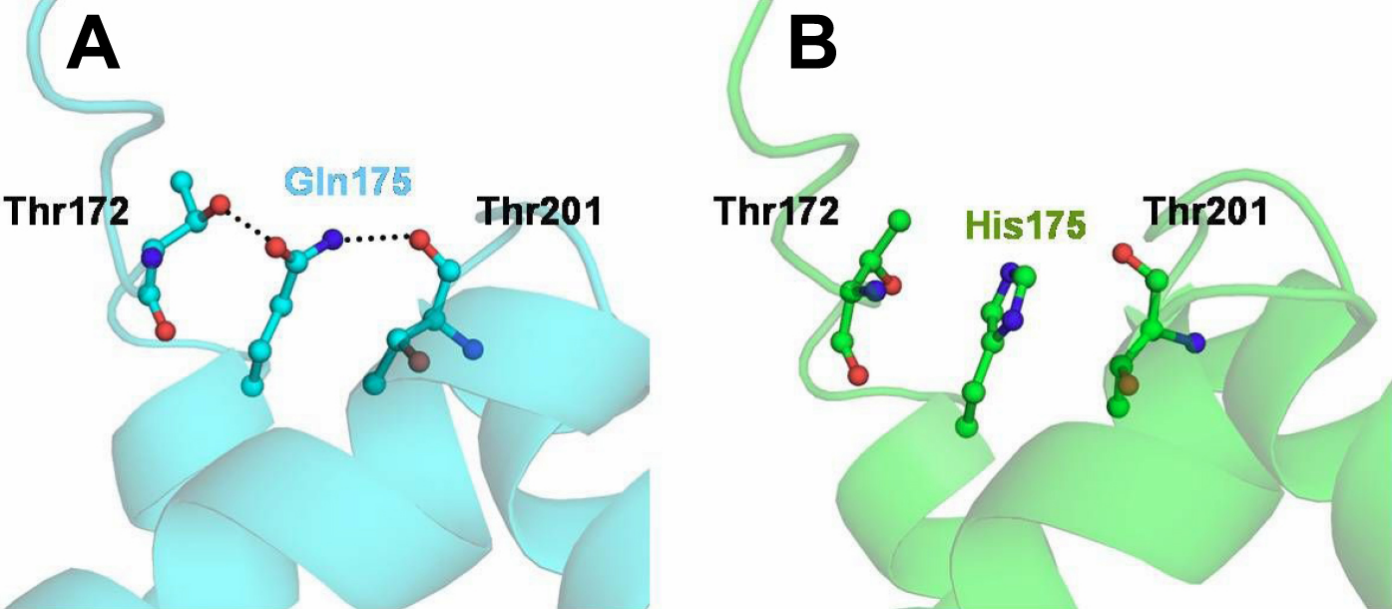
Close view of the VSX1 Q175H mutation area. The hydrogen bond interactions are marked for clarity in the wild type (**A**, cyan) and their disruption in the mutant (**B**, green) proteins.

**Figure 3:**
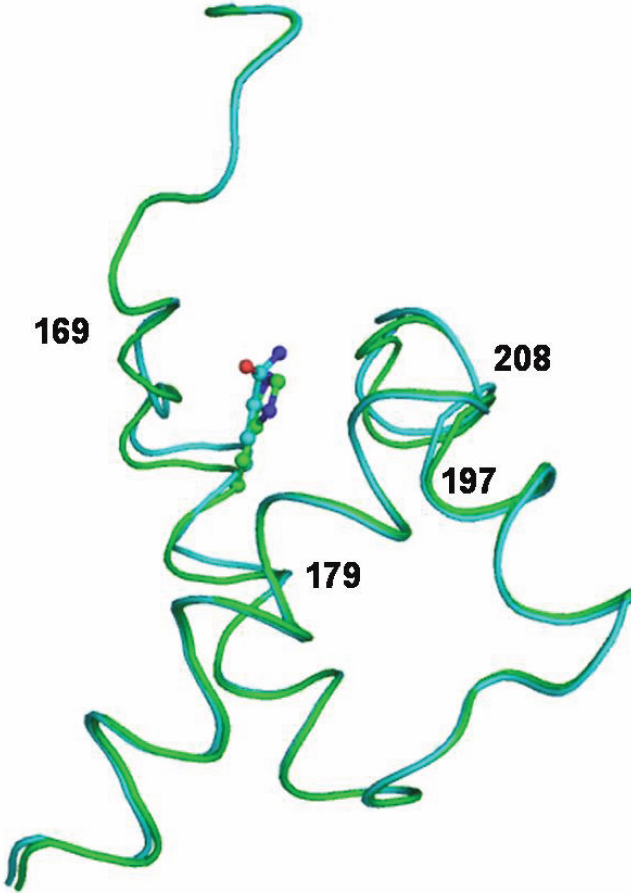
Altered loop conformations in the regions involving residues Thr169- Leu179 and Leu197 – Ile208 because of mutation in the mutant (green) as compared to wild type (cyan) proteins.

A comparison of the conformations in the wild type and mutant VSX1 revealed a variation in intermolecular interactions involving hydrogen bonds. Due to the Q175H mutation the polar glutamine residue is converted to the basic imidazole ring containing histidine which results in the complete disruption of hydrogen bonds involving the Gln175 Oε1 – Thr172 Oγ1 and Gln175 Nε2 – Thr201 O interactions in the mutant protein (Figure 2). Moreover, the Thr201, Glu178, Ala173, and Thr172 side chains were also pushed back due to the presence of a more bulky and positively charged side chain of His175 in the mutant protein. These resulting differences in the interaction due to the mutation alter the loop conformations in the regions involving residues Thr169- Leu179 and Leu197 – Ile208 (Figure 3). The threonine residue at position 201 along with neighboring residues and NH_2_-terminal residues like Thr172 are critical for specificity of DNA-binding. These conformational differences in the backbone of the molecule are localized to the regions around amino acid position 175 and the resulting changes possibly hinder the DNA/protein interactions.

## Discussion

We found a VSX1 Q175H change in the proband and his affected brother. Familial segregation of the disease revealed the mutation Q175H to be present in affected as well as unaffected family members. The molecular modeling studies showed that this mutation causes loss of molecular interactions leading to localized conformational changes in the protein affecting its DNA binding capacity proving its role in disease causation.

The resulting phenotype in turn may be dependent on the interaction of VSX1 with some modifier genes and/or the environment. Several genetic variants of *VSX1* have been described but their pathogenicity is doubtful. This gene was initially studied by Héon et al. [13] identifying the association of posterior polymorphous corneal dystrophy (PPCD) and keratoconus. One of the variants identified in their study, D144E was seen in an individual with PPCD and keratoconus and also in an individual with glaucoma without any corneal defect. The D144E mutation was initially reported as pathogenic but subsequent studies identified its presence in unaffected individuals also suggesting this to be a polymorphism [11].

Likewise there have been several studies on the genetic variants of *VSX1* but the pathogenicity could not always be confirmed as their segregation was also seen in some unaffected individuals [14].

There can be several explanations to the presence of the VSX1 Q175H change observed in our family. It could be a non-disease causing change that randomly appeared in this family in both affected (two brothers) as well as unaffected (mother) members. However its absence in 100 controls, in silico analysis [14] and molecular modeling studies suggested this to be a pathogenic change. Nevertheless, the possibility of this being a rare polymorphism still exists and screening of more controls and different populations is needed to ascertain its true nature. Another important feature is that the observed change may be pathogenic but with incomplete penetrance. The presence of this change in the unaffected mother suggests that this could be a causative mutation with incomplete penetrance.

The change can otherwise be explained based on the presence of mutation and the allergic disorder VKC– the patients may be more susceptible for KC as seen in the proband and his brother. The mother who also had the same change but did not have VKC did not develop keratoconus.The sister who had VKC but did not have mutation in *VSX1* did not manifest the disease. So, the Q175H mutation may not directly cause the disease but does so only in presence of another associated disorder. A probable explanation could be that the change in heterozygous state may not be truly pathogenic with one normal copy enough to perform its role in the individual. With changes in other genes, or the environment this single copy becomes insufficient and in turn may result in keratoconus. This can also be emphasized from the fact that in patients with LCA, mutations in the Crumbs homolog 1 (*CRB1)* gene were seen to cause keratoconus [7]. Initial reports were also based on the presence of *VSX1* mutations in patients with posterior polymorphous dystrophy (PPCD) and keratoconus, so it may be hypothesized that in presence of another associated disease condition the genetic variants of *VSX1*, or other associated genes makes the individuals more susceptible to develop keratoconus.

The studies conducted on *VSX1* either excluded or did not describe other associated abnormalities seen in their patients. These exclusions can alter the actual consequences of the variations observed in *VSX1*, as the gene and the coded protein may have a minor role by themselves but can cause significant damage in association with other genes or the environment. This explanation will even hold true for the various *VSX1* variants exhibiting low penetrance by themselves but playing a significant role in the presence of modifier genes.
